# Smallpox and Season: Reanalysis of Historical Data

**DOI:** 10.1155/2009/591935

**Published:** 2009-01-04

**Authors:** Hiroshi Nishiura, Tomoko Kashiwagi

**Affiliations:** ^1^Theoretical Epidemiology, University of Utrecht, Yalelaan 7, 3584 CL Utrecht, The Netherlands; ^2^Department of Public Health, School of Medicine, Juntendo University, 2-1-2 Hongo, Bunkyo-ku, Tokyo 113-8421, Japan

## Abstract

Seasonal variation in smallpox transmission is one of the most pressing ecological questions and is relevant to bioterrorism preparedness. The present study reanalyzed 7 historical datasets which recorded monthly cases or deaths. In addition to time series analyses of reported data, an estimation and spectral analysis of the effective reproduction number at calendar time *t*, *R*(*t*), were made. Meteorological variables were extracted from a report in India from 1890–1921 and compared with smallpox mortality as well as *R*(*t*). Annual cycles of smallpox transmission were clearly shown not only in monthly reports but also in the estimates of *R*(*t*). Even short-term epidemic data clearly exhibited an annual peak every January. Both mortality and *R*(*t*) revealed significant negative association (*P* < .01) and correlation (*P* < .01), respectively, with humidity. These findings suggest that smallpox transmission greatly varies with season and is most likely enhanced by dry weather.

## 1. Introduction

Smallpox is the only disease to have been eradicated worldwide [1]. Despite the success story of vaccination and other public health interventions, the number of susceptible individuals has grown to date following cessation of routine vaccination, and the threat of bioterrorist attack has led to debates on countermeasures in such an event [2]. Various mathematical studies have been conducted as part of a preparedness program, including large-scale simulation of a bioterrorist attack and the public health countermeasures against it [3–6]. Theoretical studies on the spread of smallpox include not only simulations but also quantitative analysis of historical data [7–10]. A statistical modeling study suggests that a small outbreak could be contained only implementing contact tracing and isolation [11]. Moreover, those who underwent vaccination in the past are believed to be still protected against severe and fatal manifestations of smallpox even today [7, 12].

Studies on smallpox control have progressed in parallel with the development of epidemiological and statistical methods, and because of the eradication before maturation of biostatistics, many questions have remained in regards to the details of the epidemiology. Seasonal variation in smallpox transmission is one of the most pressing ecological questions playing a key role in determining the transmission dynamics, should a future outbreak occur following the deliberate release [1, 4]. For example, clarification of the seasonal preference of variola virus is crucial for identifying and forecasting the disease risk using ecological data [13]. Although seasonal occurrence of smallpox was documented early on among directly transmitted infectious diseases [14, 15], and whereas the disease is believed to be one of the “winter diseases” in industrialized countries, even the presence of seasonality has not been investigated in detail.

The best available evidence stems from a series of studies by Sir Leonard Rogers (1868–1962) [16], who conducted epidemiologic surveys of smallpox outbreaks in India over a long period of time [17–19]. He also conducted a similar survey in England and Wales [20]. By analyzing the monthly mortality data from the late 19th to the early 20th century in these countries, Rogers argued that the smallpox epidemic in India is relatively uniform (i.e., not apparently cyclical) compared to that in England and Wales [17, 19, 21]. Further, he descriptively and implicitly suggested that there is a negative correlation between humidity and smallpox mortality, but there was little association between smallpox and rainfall [17, 18]. This effort was followed by Russell and Sundararajan [22] who supported the notion that a dry environment offers favorable conditions for smallpox transmission. These consistent findings have also been reported during the Smallpox Eradication Program (SEP), where a peak of smallpox incidence occurred from December to January in the Northern hemisphere (e.g., Indonesia and Bangladesh) and from May to June in the Southern hemisphere (e.g., Brazil) [23–26]. However, the observed data during the SEP were greatly modified by intensive immunizations, and perhaps because of this, epidemics in other locations were not suggested to be seasonal [26–28], leaving this issue yet to be clarified.

Despite the rigorous efforts before the global eradication, later progress on this issue was unfortunately subtle. Upham once revisited Rogers's dataset from India, anthropologically discussing potential reasons why the American Southwest was less infested by smallpox [29]. A time series technique was applied to historical data in Finland and England [30–32], showing that periodicity is mainly regulated by the susceptible fraction of a population in question [33]. However, despite the analyses on the impact of vaccination and migration on periodicity, seasonal patterns of transmission have not been explicitly studied, mainly because of a lack of data precision. In a historical study examining smallpox in England from the 16th to 17th centuries, the time referred to as the “little ice age,” it has been documented that long-term climatic changes did little to the smallpox transmission [34], but this conclusion was drawn without quantitatively and explicitly analyzing the data. Instead, the quality of time series data and its impact on seasonality were discussed in relation to social backgrounds of smallpox control [35, 36], but again no rigorous statistical analyses were made using observed data.

Accordingly, several lingering questions remain. Is smallpox transmission really seasonal? If so, is the seasonality associated with humidity? Clarification of these points will not only enhance our understanding of the pattern of smallpox transmission, but also will be crucial for identifying the seasonal preference of variola virus with some implications for bioterrorism preparedness plans. The present study is aimed at examining the presence of seasonality and clarifying the relationships between smallpox and climate. We reanalyzed various historical datasets, suggesting a new method for the analysis of time series.

## 2. Materials and Methods

### 2.1. Data Source: Historical Records

**Figure 1:**
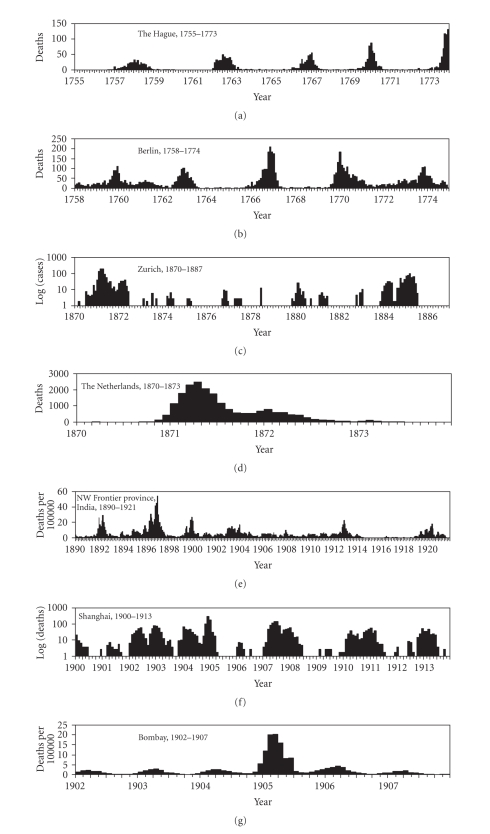
*Temporal distributions of smallpox*. Temporal patterns of smallpox are shown, which were extracted from historical records in (a) The Hague, The Netherlands, 1755–1773, (b) Berlin, Germany, from 1758–1774, (c) Zurich, Switzerland, 1870–1887, (d) the Entire Netherlands, 1870–1873, (e) Northwest Frontier province, India, 1890–1921, (f) Shanghai, China, 1900–1913, and (g) Bombay, India, 1902–1907. Death data are shown in (a), (b), (d), and (f). Cases are shown in (c). Mortality (i.e., deaths per 100 000) data are shown in (e) and (g). See [17, 39–45] for original data.

Seven temporal distributions of smallpox at different times and locations were extracted from historical literature. This literature review was based on references collected by tracking all the references given in the relevant publications and repeating this task until we could find no further references; the details are given elsewhere [37, 38]. Figure 1 shows the time series data by location with a monthly reporting interval. Chronologically, epidemic records for The Hague (1755–1773), Berlin (1758–1774), Zurich (1870–1887), the entire Netherlands (1870–1873), Northwest Frontier province in India (1890–1921), Shanghai (1900–1913), and Bombay (1902–1907) provide monthly data of smallpox with time and were used for further analysis [17, 39–45]. The first two records contain data before the introduction of vaccination. Except for Zurich, which documents the monthly number of cases, the remaining datasets record only monthly deaths. Death data are given as the absolute number of deaths, except where indicated. With regard to the magnitude of the epidemics, the annual averages of the disaster size were 10.1 deaths (The Hague), 32.9 deaths (Berlin), 9.9 cases (Zurich), 428.6 deaths (the entire Netherlands), 5.28 deaths per 100 000 (Northwest Frontier province in India), 21.5 deaths (Shanghai), and 2.45 deaths per 100 000 (Bombay). By examining another historical record of the smallpox epidemic in Tokyo, it was found that the mean (and the standard deviation) and the median (25–75% quartile) time from onset to death were 29.1 (13.8) and 26.0 (19.0–37.0) days, respectively [46]. Thus, it is reasonable to assume that the relative frequency of death with time represents that of onset accompanied by approximately a 1 month delay. Moreover, the infection may have happened approximately half a month before the onset [9]. Meteorological variables with time were given only in Rogers's observations [17], which contained the monthly rainfall (inch) and the absolute humidity.

### 2.2. Time Series Analysis

First, the presence of seasonality was examined for all 7 datasets using spectral density analysis. Spectral analysis is based on the idea of a theoretical power-spectrum, which partitions the total variance of the series among sinusoidal components [47]. In other words, spectral density decomposes a time series function into a sum of sines and cosines. The density plot (i.e., correlogram) was graphically plotted to determine if a sharp peak at a period of 12 months exists, corresponding to an annual cycle (i.e., seasonality).

### 2.3. Estimation of the Effective Reproduction Number

Second, seasonality that was evaluated using the effective reproduction number, *R*(*t*), defined as the actual average number of secondary cases per primary case at calendar time *t*. *R*(*t*) shows a time-dependent variation with a decline in susceptible individuals (intrinsic factors) and with the implementation of control measures (extrinsic factors). If *R*(*t*) < 1, it suggests that the epidemic is in decline (vice verca, if *R*(*t*) > 1). This approach was employed to clearly show the seasonal patterns of transmission and to partly address the issue of dependence among cases, that is, statistically, the observation of an infected individual is not independent of other infected individuals, since the disease is transmitted directly from human to human.

The following approximation was made to derive estimates of *R*(*t*). Supposing that the number of new infections at calendar time *t* is *I*(*t*), the transmission dynamics are described by the renewal equation using *R*(*t*) [48, 49]:

(1) $$I(t)=R(t)\int_{0}^{\infty }I(t-\tau )\omega (\tau )d\tau ,$$

where *ω*(*τ*) is the probability density function of the generation time. The right-hand side of (1) represents secondary transmissions at calendar time *t*, which are determined by the number of those who were infected at time *t* − *τ*, *I*(*t* − *τ*), and the magnitude of secondary transmissions at time *t*, *R*(*t*). Since the data in the present study were recorded only monthly, the equation has to be simplified to comply with discrete points of time data. From the beginning of the history of mathematical modeling of smallpox in the late 19th century [50], cases tended to be modeled by generation, the idea of which is applied as follows. Given the number of cases in generation *i*, *I*_*i*_, the expected number of cases in generation *i* + 1, E(*I*_*i*+1_) is given by

(2) $$\text{E}(I_{i+1})=R_{i}I_{i},$$

where *R*_*i*_ is the effective reproduction number of generation *i* [51]. That is, the reproduction number is simply given by ratio of successive generations of cases, which was implicitly understood in history by a pioneering epidemiologist, Clare Oswald Stallybrass (1881—1951) who applied the theory to analyze the seasonality of various infectious diseases [52, 53]. Since the mean generation time of smallpox is approximately 15 days (i.e., half a month) [50, 54], monthly data contains exactly two generations. Let us consider three successive generations, *i*, *i* + 1, and *i* + 2. Given the reproduction numbers *R*_*i*_ and *R*_*i*+1_, we get

(3) $$\begin{array}{c} \text{E}(I_{i+1})=R_{i}I_{i},\\ \text{E}(I_{i+2})=R_{i+1}I_{i+1}. \end{array}$$

Considering that the generations *i* and *i* + 1 are grouped together and reported in the same month *j*, the reproduction number cannot be estimated by generation *i*. Instead, by assuming that the reproduction numbers in the successive generations are identical, that is, *R*_*i*_ = *R*_*i*+1_(:= *R*_*j*_), (3) can be rearranged as

(4) $$\begin{array}{c} \text{E}(I_{i+1})=R_{j}I_{i},\\ \text{E}(I_{i+2})=R_{j}I_{i+1}. \end{array}$$

The expected number of cases in the next generation *i* + 3 is given by product of *I*_*i*+2_ and the reproduction number in the next month *j* + 1, *R*_*j*+1_, that is,

(5) $$\text{E}(I_{i+3})=R_{j+1}I_{i+2}.$$

Given (4) and (5), the number of cases in month *j* + 1, *C*_*j*+1_(:= *I*_*i*+2_ + *I*_*i*+3_) is given using *C*_*j*_(:= *I*_*i*_ + *I*_*i*+1_), that is,

(6) $$\begin{array}{ll} \text{E}(C_{j+1}) & =E(I_{i+2}+I_{i+3})\\ & =(1+R_{j+1})I_{i+2}\\ & =(1+R_{j+1})R_{j}I_{i+1}\\ & =(1+R_{j+1})R_{j}^{2}I_{i}\\ & =(1+R_{j+1})R_{j}^{2}\frac{C_{j}}{1+R_{j}}. \end{array}$$

We assume that the expected values are sufficient to characterize Poisson distributions. This assumption indicates that the conditional distribution of the reported number of cases in month *j* + 1, *C*_*j*+1_, given *C*_*j*_ is given by

(7) $$C_{j+1}\;|\;C_{j}∼\text{Poisson}\left[\frac{(1+R_{j+1})R_{j}^{2}}{1+R_{j}}C_{j}\right].$$

Thus, for the observation of cases (or deaths with a 1 month lag) from month 0 to *N*, the likelihood of estimating *R*_*j*_ is given by

(8) $$\begin{array}{ll} L & =\text{constant}\times \overset{N-1}{\underset{j=0}{\prod }}{\left[\frac{(1+R_{j+1})R_{j}^{2}}{1+R_{j}}C_{j}\right]}^{C_{j+1}}\\ & \;\times \exp\;\left[-\frac{(1+R_{j+1})R_{j}^{2}}{1+R_{j}}C_{j}\right]. \end{array}$$

By minimizing the negative logarithm of (8), the maximum likelihood estimates of the monthly-approximated reproduction numbers, *R*_*j*_ were obtained.

### 2.4. Multivariate Modeling

Third, to identify the characteristic factors of seasonal variation in smallpox transmissions, the relationships between meteorological variables (i.e., rainfall and humidity) and incidence (mortality) as well as the effective reproduction number were examined. To examine the influence of seasonal variables on the temporal trend of smallpox, we employed one of the generalized linear models with the construction of a Poisson regression model incorporating monthly and yearly terms [55]:

(9) $$\begin{array}{ll} \text{E}(C_{j}) & =\exp\{\alpha +\beta_{1}(\text{year})\\ & \;\;\;+\beta_{2}\left[\sin\;\left(2\pi \times \frac{\text{month}}{12}\right)\right]\\ & \;\;\;+\beta_{3}\left[\cos\;\left(2\pi \times \frac{\text{month}}{12}\right)\right]\}, \end{array}$$

where $\begin{array}{c} \text{E} \end{array}$(*C_j_*) is the expected number of cases (deaths) in month *j*, *α* is a constant, and *β*_*i*_ are the regression coefficients for year or month. The relationship was investigated using both univariate and multivariate models. In the multivariate model, the year of occurrence was controlled for, but the sine and cosine of the month were not included due to colinearity with rainfall. The mortality rate ratios (MRR) for the occurrence of smallpox death were used to evaluate the impact of each meteorological variable on smallpox.

With regard to the relationship between meteorological variables and *R*(*t*), multiple linear regression analysis was employed to determine factors contributing to *R*(*t*). Because of the obvious cyclical nature of the observed data yielding an autocorrelation in the linear regression analysis (Durbin-Watson = 0.23), the monthly periodic terms (as shown in (9)) were added to the list of independent variables. The level of statistical significance was set at *α* = 0.05. All statistical data were analyzed using the statistical software JMP version 7.0 (SAS Institute Inc., Cary, NC, USA).

## 3. Results

### 3.1. Temporal Distribution and Spectral Density

**Figure 2:**
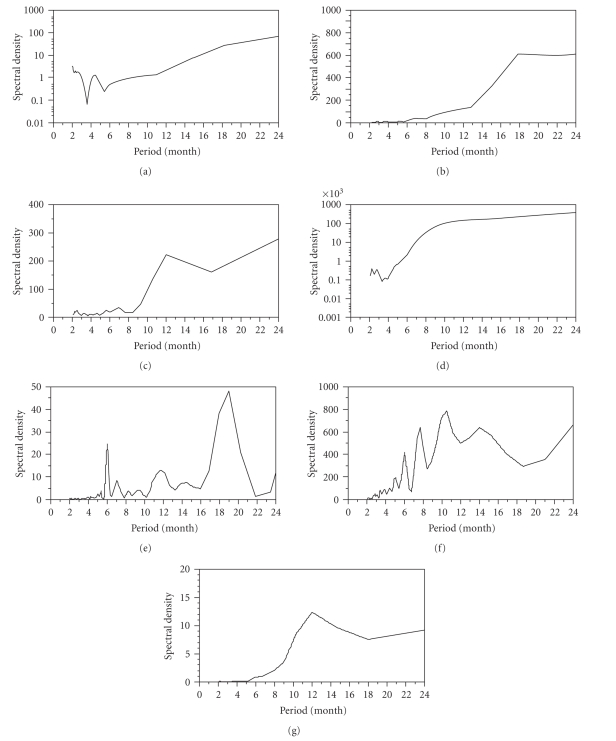
*The spectral density plots for smallpox occurrences*. (a)–(f) correspond to locations as chronologically ordered in Figure 1. A sharp peak at a period of 12 months corresponds to the annual cycle (seasonality), while other longer peaks may reflect a super-annual cycle (periodicity). No adjustment was made in drawing the plots.

The spectral densities are shown in Figure 2 which can be reasonably interpreted by comparatively examining the temporal distributions (Figure 1). With regard to the data collected from The Hague and Berlin, the observations of which were made before the introduction of vaccinations, periodic epidemics (i.e., super-annual cycles) are apparent where the interepidemic period ranges from 3 to 5 years (see Figures 1(a) and 1(b)). However, the annual cycle is not seen, and thus, the spectral densities do not show a clear seasonal pattern (Figures 2(a) and 2(b)). On the contrary, the time series data in Zurich and Shanghai clearly revealed a peak at 12 months (Figures 2(c) and 2(f)). The entire Netherlands data covers a relatively short period of time compared to the other datasets (Figure 1(d)) with unclear seasonal and periodic frequencies in the spectral diagram (Figure 2(d)). Although a small peak is seen at 12 months for the data in the Northwest Frontier province in India (Figure 2(e)), the density plot exhibits a multimodal pattern, reflecting an irregular temporal distribution (Figure 1(e)). In the Bombay data, the annual cycle is most clearly highlighted in the temporal distribution (Figure 1(g)), which is also reflected in the spectral density (Figure 2(g)).

### 3.2. Effective Reproduction Number

**Figure 3:**
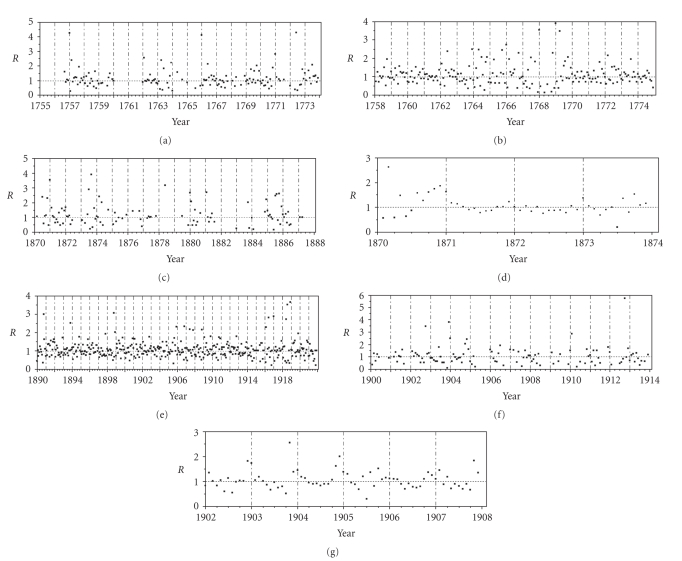
*Estimates of the effective reproduction numbers with time, *R*(*t*)*. (a)–(f) correspond to locations as chronologically ordered in Figure 1. The horizontal dashed line indicates where the reproduction number is unity. The vertical broken lines represent every January. *R*(*t*) cannot be estimated where the observed number of cases (or deaths) was 0 and is not shown for such time points.

**Figure 4:**
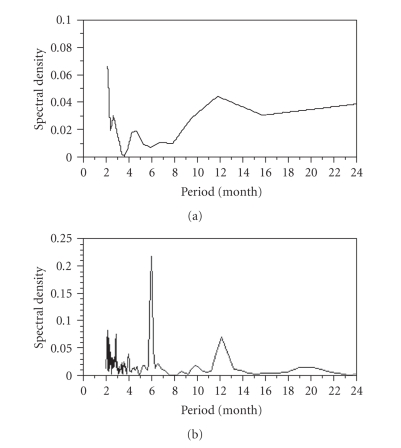
*The spectral density plots for *R* of smallpox*. The spectral density plots for the estimated reproduction numbers of smallpox in (a) the entire Netherlands, 1870–1873 and (b) Northwest frontier province, India, 1890–1921. Compared with Figures 2(d) and 2(e), the spectral densities clearly indicate a sharp peak at a period of 12 months.

Figure 3 plots estimates of the effective reproduction number as a function of calendar time. The vertical broken lines represent January in every year, while a horizontal dashed line is a reference value yielding *R*(*t*) = 1, that is, the threshold condition of an epidemic. *R*(*t*) tends to increase during the winter season for three early records (Figures 3(a), 3(b), and 3(c)), but the annual cycles are not seen. However, the short-term epidemic data for the entire Netherlands clearly shows that three peaks of *R*(*t*) coincide in every January with estimates above unity (Figure 3(d)). A similar pattern is observed in Shanghai and Bombay (Figures 3(f) and 3(g)). Figure 4 shows the spectral density plots of *R*(*t*) for the entire Netherlands and Northwest Frontier province in India. Although spectral densities of death and mortality (Figures 2(d) and 2(e)) did not exhibit a clear annual cycle, the obvious peak at 12 months is seen for both datasets in terms of *R*(*t*) (Figures 4(a) and 4(b)). That is, seasonal patterns of smallpox transmission were reasonably shown with the use of *R*(*t*) even for the short- and long-term time series.

### 3.3. Factors Characterizing Seasonal Variation

Table 1 shows the output of univariate and multivariate models for explaining smallpox mortality in India using meteorological variables. In both models, rainfall was not significantly associated with smallpox mortality. However, significant negative association was found for humidity (adjusted MRR = 0.387 (95% confidence interval (CI): 0.311, 0.481), *P* < .01). Table 2 summarizes the relationship between the effective reproduction number and meteorological variables using a multiple linear regression model. On a whole, the model showed a weak predictive ability. However, humidity was again identified as an explanatory variable which significantly reduces the effective reproduction number (*P* < .01). No significant correlation was found between *R*(*t*) and rainfall.

## 4. Discussion

The present study reanalyzed historical records of smallpox to examine the presence of seasonality and to partly clarify the characteristic factors. Although 18th century data did not show an apparent annual cycle, the remaining records reasonably showed seasonal variations either in the monthly observation or the reproduction number. In particular, even the short-term epidemic data for the entire Netherlands clearly revealed peaks of transmission every January. Although several important meteorological variables were missing (e.g., temperature and atmospheric pressure), Rogers's observation permitted investigations of a few variables as underlying factors characterizing the seasonality. Analyzing the meteorological data in India, both smallpox mortality and the reproduction number yielded significant negative association and correlation with humidity. Rainfall did not appear to be a useful predictor of seasonality.

One important message drawn from this exercise is that smallpox transmission is confirmed as seasonal and this is most likely associated with dry weather. This finding is consistent with implicit suggestions which have accumulated in the historical literature [1, 17, 19]. Whereas the data from The Hague and Berlin did not offer the relevant interpretations, their periodic peaks were also observed during the winter seasons. Assuming that these records captured mainly the large periodic outbreaks alone, it is plausible that the old data were accompanied by underreporting during less intensive years, and thus, did not precisely contain subtle seasonal fluctuations. Given that the seasonal force of infection was obvious even in the short-term epidemic data from the entire Netherlands, not only endemic but also epidemic smallpox would greatly vary with the season and most likely would be enhanced by dry weather. Historically, virologists attempted to attribute the annual cycle to the seasonal preference of the variola virus [56–58]. To date, it is known that the variola virus could survive in an infective state under different conditions of temperature and humidity [56, 57]. However, as temperature and humidity rise above 30°C and 55%, respectively, the virus is known to immediately lose infectivity [57]. Such a virological explanation supports the epidemiologic findings from this present study and reasonably explains the seasonal preference of the virus as a factor behind the seasonality of outbreaks. The above-mentioned point implies that we cannot ignore the seasonality even in the event of a short-term reintroduction of variola virus due to bioterrorist attack.

A technical development in analyzing seasonal data is also notable. Since the observation of an infected individual is not independent of other infectious individuals, direct application of the generalized linear model has not been appropriate to date. One approach to resolve this issue is to employ a Bayesian method, explicitly dealing with dependence in a Poisson regression model [59], which is, however, computationally complicated for nonspecialists. As an alternative, the present study suggested the use of *R*(*t*). *R*(*t*) reasonably reflects time-dependent changes in the susceptible fraction of the population in question and other various factors determining the transmission (including seasonality) [60, 61]. In particular, it should be noted that *R*(*t*) does not reflect onset or death but can represent an infection event with time, proving its potential as a marker to model seasonal and periodic transmission cycles. In addition, quantitative assessment of *R*(*t*) is theoretically important, because the amplitude of seasonal forces of infection characterizes the length of the interepidemic period [33, 62, 63]. A continued super-annual cycle mathematically requires seasonally varying forces of infection, which determines the season-specific threshold condition [64] and evolutionary dynamics of a disease [65, 66]. To the best of our knowledge, the present study is the first to suggest a quantitative method to reasonably extract the amplitude using the notation of *R*(*t*) and extending the previous efforts of Stallybrass [53].

Most infectious diseases show characteristic seasonal variations in incidence. The present study confirms that the transmission of smallpox does vary with season. However, compared to the seasonal ecology of insects in vector-borne diseases, seasonal factors for directly transmitted diseases are far more complex, and thus, questions remain as to what exactly are the factors behind the seasonality of smallpox. At least, experimental evidence supports the role of dry weather in the dynamics of influenza [67, 68]; a recent study found that low (dry) relative humidity in the range of 20 to 30% produced the spread of the influenza virus faster than at relative humidity in higher percentages. In fact, at a humidity of 80% or above, the study found no spread of the flu [68]. Since there are also various social factors which vary with the season, the seasonal preference of pathogens cannot be captured without sufficiently highlighting the time-varying human contact patterns, and thus, further analyses (e.g., reanalysis of small-scale outbreaks where we can adjust the contact frequency) are needed. We hope that the present study enhances the similar reanalysis of historical data, triggering an interest in investigating the relationship between the transmission of directly transmitted infectious diseases and climatic changes.

## 5. Conclusions

Seven historical datasets of smallpox were reanalyzed to examine the presence of seasonality and to identify the characteristic factors. Annual cycles were clearly shown not only in the monthly reports but also in the estimates of the effective reproduction number. Even for a short-term epidemic, the transmission of smallpox would most likely be enhanced by dry weather.

## Figures and Tables

**Table 1:** Monthly weather patterns and smallpox mortality in Northwest frontier province, India, from 1890–1921.

Meteorological element	Univariate model		Multivariate model^¶^	
	MRR^‡^(95% CI^†^)	*P*	MRR^‡^(95% CI^†^)	*P*
Rainfall (inch)	0.979 (0.952, 1.003)	.10	0.991 (0.964, 1.018)	.53
Absolute humidity	0.384 (0.309, 0.477)	<.01	0.387 (0.311, 0.481)	<.01

^‡^MRR, mortality rate ratio, reflects change in risk of smallpox death per unit (or absolute value) in the meteorological variable in question. ^†^CI, confidence interval. ^¶^The multivariate model was also adjusted for the calendar year.

**Table 2:** Monthly weather patterns and the effective reproduction number of smallpox in Northwest frontier province, India, from 1890–1921.

Variable	*β* ^†^	S.E.^‡^	*t*-Ratio	*P*
Intercept	1.579	0.139	11.37	<.01
Rainfall (inch)	0.008	0.015	0.51	.61
Absolute humidity	−1.169	0.319	−3.66	<.01
Cosine	−0.181	0.078	−2.32	.02
Sine	−0.211	0.064	−3.29	<.01

^†^Parameter coefficient; ^‡^Standard error; *r* ^2^ = 0.057 (*F*-ratio = 5.67, *P* < .01), dependent variable = effective reproduction number, Durbin-Watson = 2.600.
